# Early impact of the coronavirus disease (COVID-19) pandemic and physical distancing measures on routine childhood vaccinations in England, January to April 2020

**DOI:** 10.2807/1560-7917.ES.2020.25.19.2000848

**Published:** 2020-05-14

**Authors:** Helen I McDonald, Elise Tessier, Joanne M White, Matthew Woodruff, Charlotte Knowles, Chris Bates, John Parry, Jemma L Walker, J Anthony Scott, Liam Smeeth, Joanne Yarwood, Mary Ramsay, Michael Edelstein

**Affiliations:** 1 NIHR Health Protection Research Unit (HPRU) in Immunisation, London, United Kingdom; 2 Department of Infectious Disease Epidemiology, London School of Hygiene and Tropical Medicine, London, United Kingdom; 3 Immunisation and Countermeasures Division, Public Health England, Colindale, United Kingdom; 4 The Phoenix Partnership (TPP) (Leeds) Ltd, Leeds, United Kingdom; 5 Statistics, Modelling and Economics Department, Public Health England, Colindale, United Kingdom

**Keywords:** COVID-19, pandemics, Measles-Mumps-Rubella vaccine, DTaP-IPV-Hib-HBV vaccine

## Abstract

Using electronic health records, we assessed the early impact of coronavirus disease (COVID-19) on routine childhood vaccination in England by 26 April 2020. Measles-mumps-rubella vaccination counts fell from February 2020, and in the 3 weeks after introduction of physical distancing measures were 19.8% lower (95% confidence interval: −20.7 to −18.9) than the same period in 2019, before improving in mid-April. A gradual decline in hexavalent vaccination counts throughout 2020 was not accentuated by physical distancing.

Childhood vaccination coverage in the United Kingdom (UK) is routinely monitored quarterly, but more timely monitoring is required during the disruption of a pandemic [1,2]. We analysed electronic patient records from primary care to describe changes in delivery of first doses of hexavalent vaccine (against diphtheria, tetanus, pertussis, polio, *Haemophilus influenzae* type b and hepatitis B) and measles-mumps-rubella (MMR) vaccine as part of the routine childhood vaccination programme in England during the coronavirus disease (COVID-19) outbreak until 26 April 2020 (weeks 1 to 17).

## Near real-time records of routine childhood vaccinations from primary care

Two key milestones in the routine childhood immunisation programme delivered in primary care are first universal vaccinations at the age of 8 weeks, which include the hexavalent vaccine, and vaccinations at the age of 1 year, which include the first dose of MMR vaccine [3].

Aggregated weekly counts of the first hexavalent vaccinations delivered to infants younger than 6 months and of the first MMR vaccinations delivered to children aged 12 to 18 months were provided from The Phoenix Partnership (TPP) SystmOne for the first 17 weeks of 2019 and 2020. SystmOne is a software system which provides electronic patient records for more than 2,600 primary care practices in the UK and more than 35 child health providers [4]. Age ranges were selected to describe vaccinations delivered as part of the routine vaccination programme rather than catch-up campaigns, and match age ranges for national routine surveillance of vaccine coverage [1]. Data were anonymous throughout, having been originally extracted as aggregated weekly vaccination counts for the purpose of SystmOne data checks which use population level data.

To minimise changes in denominator, only providers active in SystmOne since 2018 contributed to the dataset. The majority of vaccinations entered into SystmOne are entered by general practices in real time. However, vaccinations delivered in general practices which use other patient record software may be recorded at a delay into the SystmOne integrated patient record by local Child Health systems co-ordinating vaccination scheduling. To avoid artefacts from lags in data recording, we included only vaccinations recorded on the same day as they were delivered (which comprised more than 70% of the total hexavalent and MMR vaccination doses recorded as delivered in weeks 1 to 17 of 2019).

For weeks 1 to 17, the dataset included 69,568 hexavalent doses delivered in 2019 and 67,116 in 2020 as well as 68,849 MMR doses delivered in 2019 and 66,301 in 2020.

## Ethical statement 

This analysis was conducted as part of public health usual practice, and was not conducted for research. Ethics approval was therefore not sought.

## How did vaccination counts change during the COVID-19 pandemic?

**Figure 1:**
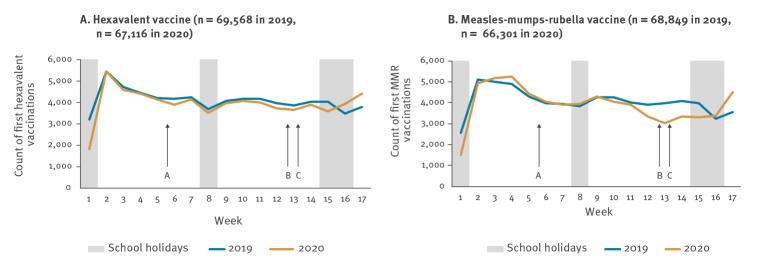
Hexavalent and measles-mumps-rubella weekly vaccination counts, England, weeks 1–17, 2019 and 2020

Hexavalent vaccination counts followed a similar pattern in 2020 as in 2019, varying week by week; particularly low counts in week 1 of both years are probably explained by holidays (Figure 1). The MMR vaccination counts also followed a similar pattern in 2020 until week 11, when they fell, and remained low for several weeks before rising again in weeks 16 and 17.

Arrows indicate events in 2020: A: World Health Organization declares Public Health Emergency of International Concern on 30 January (week 5). B and C: Successive introduction of physical distancing measures in the United Kingdom on 20 and 23 March (weeks 12 and 13).

The percent change in vaccinations counts in 2020 compared with 2019 varied over the course of the COVID-19 pandemic (Table). At the start of 2020 (weeks 1 to 9), hexavalent vaccination was 5.8% lower (95% confidence interval (CI): −6.0 to −5.5) and MMR vaccination 1.0% lower (95% CI: −1.1 to −0.9) compared with 2019.

**Table:** Hexavalent and measles-mumps-rubella vaccination counts in 2020 compared with 2019, by week, England, weeks 1–17

Week	Hexavalent vaccine first dose			MMR vaccine first dose		
	2019 n	2020 n	Percent change % (95% CI)^a^	2019 n	2020 n	Percent change % (95% CI)^a^
1	3,191	1,835	−42.5 (−45.2 to −39.8)	2,564	1,515	−40.9 (−43.9 to −38.0)
2	5,447	5,441	−0.1 (−0.2 to 0.0)	5,098	4,935	−3.2 (−3.7 to −2.7)
3	4,720	4,591	−2.7 (−3.2 to −2.3)	5,005	5,170	3.3 (2.8 to 3.8)
4	4,426	4,418	−0.2 (−0.3 to −0.1)	4,893	5,246	7.2 (6.5 to 8.0)
5^b^	4,185	4,134	−1.2 (−1.6 to −0.9)	4,292	4,445	3.6 (3.0 to 4.1)
6	4,168	3,895	−6.5 (−7.4 to −5.7)	3,988	4,029	1.0 (0.7 to 1.3)
7	4,229	4,114	−2.7 (−3.2 to −2.2)	3,940	3,920	−0.5 (−0.7 to 1.3)
8	3,676	3,497	−4.9 (−5.6 to −4.1)	3,839	3,943	2.7 (2.1 to 3.2)
9	4,051	3,974	−1.9 (−2.3 to −1.5)	4,241	4,278	0.9 (0.6 to 1.2)
10	4,151	4,075	−1.8 (−2.2 to −1.4)	4,262	4,057	−4.8 (−5.5 to −4.1)
11	4,180	3,981	−4.8 (−5.4 to −4.1)	4,010	3,906	−2.6 (−3.1 to −2.1)
12^b^	3,967	3,704	−6.6 (−7.5 to −5.8)	3,905	3,332	−14.7 (−16.0 to −13.4)
13^b^	3,855	3,657	−5.1 (−5.9 to −4.4)	3,990	3,024	−24.2 (−25.9 to −22.5)
14	4,039	3,882	−3.9 (−4.5 to −3.3)	4,079	3,345	−18.0 (−19.4 to −16.6)
15	4,030	3,591	−10.9 (−12.0 to −9.8)	3,975	3,290	−17.2 (−18.6 to −15.8)
16	3,469	3,912	12.8 (11.7 to 13.9)	3,227	3,356	4.0 (3.3 to 4.7)
17	3,784	4,415	16.7 (15.5 to 17.9)	3,541	4,510	27.4 (25.9 to 28.8)
**Total**	69,568	67,116	−3.5 (−3.7 to −3.4)	68,849	66,301	−3.7 (−3.8 to −3.6)

CI: confidence interval; MMR: measles-mumps-rubella.

^a^ 95% CI calculated using the Wilson method.

^b^ Events in 2020 included: World Health Organization declares Public Health Emergency of International Concern on 30 January (week 5); and successive introduction of physical distancing measures in the United Kingdom on 20 and 23 March (weeks 12 and 13).

Weeks 10 to 12 were a transition period, with public discussion of physical distancing from at least week 10 [5]. On 12 March (week 11), the UK government advised that anyone with a new continuous cough or a fever should self-isolate for 7 days. Physical distancing measures were introduced nationally on 20 March (end of week 12) and subsequently extended on 23 March (start of week 13), closing schools and requiring everyone in the UK to avoid gatherings and non-essential use of public transport, limit contact with others and work from home if possible [6]. In weeks 10 to 12 of 2020, hexavalent vaccination was 4.4% lower (95% CI: −4.8 to −4.0) and MMR vaccination 7.2% lower (95% CI: −7.7 to −6.7) than in 2019.

In the 3 weeks after introduction of full physical distancing measures (weeks 13 to 15), hexavalent vaccination was 6.7% lower (95% CI: −7.1 to −6.2) and MMR vaccination 19.8% lower (95% CI: −20.7 to −18.9) than in 2019. Although physical distancing measures remained unchanged nationally throughout the rest of the study period, vaccination counts were higher in weeks 16 and 17 of 2020 than for the same weeks in 2019, for both vaccines.

**Figure 2:**
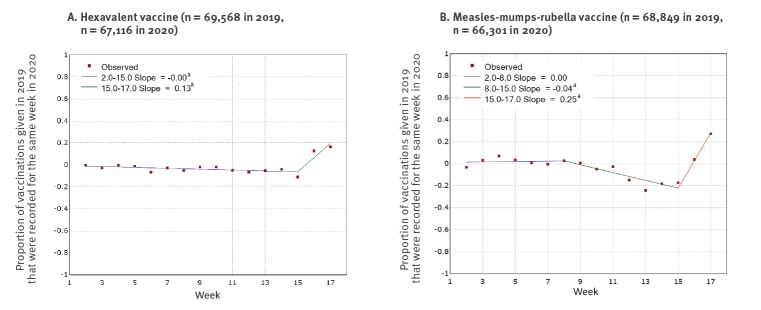
Percent change in hexavalent and measles-mumps-rubella vaccination in 2020 compared with 2019, by week^a^, England, weeks 1–17

^a^ Week 1 excluded as anomalous (due to national holidays) and not comparable between 2019 and 2020. Routine vaccination appointments would not typically be expected until after the New Year’s bank holiday, which fell on a Tuesday of in 2019 and Wednesday in 2020 and so would comprise 3 working days in 2019 and 2 working days in 2020.

Trends over time in the percent change of vaccination counts in 2020 compared with 2019 were modelled using Joinpoint regression (version 4.8.0.0), which finds the best fit for points of change in trend [7]. For the hexavalent vaccination, this suggested a general decrease in vaccination throughout weeks 1 to 15 in 2020 compared with the same weeks in 2019, which did not accentuate on introduction of physical distancing, but reversed in week 15, with a percent increase in weeks 16 and 17 of 2020 compared with 2019 (Figure 2). The percent change of first MMR doses delivered in 2020 compared with 2019 was steady until week 9, but then decreased to a low point of −24.2% (95% CI −25.9 to −22.5%) in week 13 before also reversing, with a percent increase in weeks 16 and 17 of 2020 compared with 2019 (Figure 2).

## How did changes in vaccination counts vary geographically?

**Figure 3:**
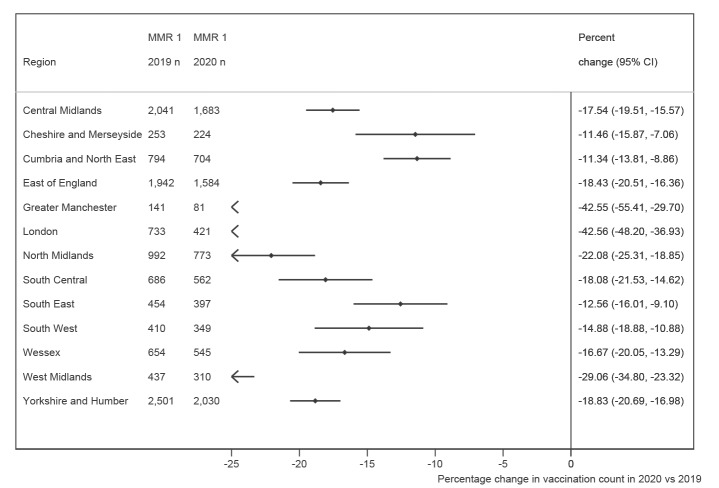
Percent change in MMR vaccination counts in the first three weeks of physical distancing (weeks 13 to 15) of 2020 compared with 2019, by region

In the 3 weeks following introduction of physical distancing measures (weeks 13–15), the percent change in hexavalent vaccination in 2020 compared with 2019 varied by region, ranging from increases of +17.4% (95% CI: 12.4 to 22.4) in Cheshire and Merseyside to decreases of more than −10% in Greater Manchester, London, the West Midlands and Yorkshire and the Humber. In contrast, MMR vaccination during weeks 13 to 15 was lower in 2020 than 2019 for all regions. The size of the percent decrease varied, with the greatest falls in London, Greater Manchester, and the West Midlands (Figure 3). By week 17 of 2020, the percent change in vaccination counts in 2020 compared with 2019 had improved in all regions, but only two regions had reached the cumulative vaccination count seen in week 17 of 2019.

CI: confidence interval;

## Discussion

MMR vaccination started falling in 2020 before introduction of physical distancing measures implemented in response to the COVID-19 epidemic. In the first 3 weeks of physical distancing, MMR vaccination counts were 19.8% lower (95% CI: −20.7 to −18.9) than for the same period in 2019. There was a general decrease in hexavalent vaccinations delivered in 2020 compared with 2019, but no evidence of an increase in the rate of decline with the introduction of physical distancing measures. Counts of both vaccinations increased in weeks 16 and 17, despite physical distancing measures remaining in place.

One plausible explanation is that COVID-19 messaging about staying home initially overwhelmed the message that the immunisation programme was to remain operating as usual. In England, this appears to have affected MMR vaccination more than primary infant vaccinations, and qualitative work is underway to explore the reasons for this. The message to continue routine immunisation programmes became more visible when the Joint Committee for Immunisation and Vaccination published a statement in week 16, on 17 April [8]. The relative increase in week 16 of 2020 compared with 2019 could partly be attributed to a low vaccination count in 2019 because of school holidays – a similar decrease was seen in the same week in 2018 and 2017. This effect may have been less relevant in 2020 when schools were closed from week 13 and it was not possible to go on holidays because of the lockdown. However, a percent increase was also observed in week 17 for both vaccinations. This is promising for an early recovery in vaccination delivery following the encouragement to continue vaccinating, but will need monitoring to ensure it is sustained.

Our findings are consistent with reports of decreased vaccine counts in other high-income countries [9,10]. In particular, in the United States, routine paediatric vaccine counts decreased after a national emergency was declared on 13 March, and have recovered somewhat for measles-containing vaccinations to children younger than 2 years, which the authors suggest could reflect promotion of childhood vaccinations in the context of the pandemic for this age-group [11]. 

National physical distancing guidance applied across all regions, but the impact of the pandemic and physical distancing may vary by region, either as a result of a varying burden of disease (reducing healthcare capacity or preventing attendance), behavioural change (including physical distancing measures) or local initiatives to enable vaccination to continue. The greatest percent decrease in MMR vaccination in weeks 13 to 15 was seen in London, which had a high burden of COVID-19, but decreases in MMR vaccination were seen across all regions in this period, including regions with a low incidence of COVID-19 infection, suggesting that the changes were not solely due to COVID-19 infection burden. SystmOne use varies regionally but it is unlikely that software system choice would result in vaccination counts being unrepresentative within regions [12]. 

A limitation of this analysis is that changing counts of delivered vaccinations could be driven by numbers of eligible infants rather than vaccine coverage. Decreasing birth rates may plausibly explain the overall reduction in counts of both vaccines in 2020 compared with 2019 [13], and migration could play a role too, but these cannot explain the size and timing of the changes in MMR vaccination during weeks 10 to 17 of 2020. Deferral of data entry could explain some decrease in real-time vaccination counts, but not the subsequent increase. Comparison of vaccination counts in 2020 to 2019 could also be affected by any changes in vaccine coverage in 2019. National quarterly surveillance of hexavalent and first MMR vaccine coverage from 2017 to 2019 suggests that 2019 was unexceptional [2]. Over the long term, there has been a gradual but steady decline in childhood vaccine coverage, with a total decrease of ca 2% over the past 5 years for both vaccines [1]. While it is plausible that this trend has continued in 2020, the speed and magnitude of changes seen with MMR doses cannot be explained by these trends.

## Conclusion

It is vital that routine childhood vaccinations are timely, particularly for diseases such as measles for which a high coverage is required to prevent outbreaks [8,14]. The Regional Office for Europe of the World Health Organization has advised that routine immunisation services should continue to aim for high population immunity [15]. Countries will require immunisation recovery plans with innovative approaches to delivery that maintain physical distancing requirements. We report the impact of COVID childhood vaccinations delivered in primare care, but other vaccinations which are routinely delivered in schools (HPV, MenACWY, Td/IPV) have been interrupted by school closures, and will also require catch-up programmes. Continuous and timely assessment of vaccine coverage will be required to respond to potentially volatile changes during the COVID-19 pandemic.
